# Modulators of Prostate Cancer Cell Proliferation and Viability Identified by Short-Hairpin RNA Library Screening

**DOI:** 10.1371/journal.pone.0034414

**Published:** 2012-04-11

**Authors:** Kimberly Brown Dahlman, Joel S. Parker, Tambudzai Shamu, Haley Hieronymus, Caren Chapinski, Brett Carver, Kenneth Chang, Gregory J. Hannon, Charles L. Sawyers

**Affiliations:** 1 Human Oncology and Pathogenesis Program, Sloan-Kettering Cancer Center, New York, New York, United States of America; 2 Department of Urology, Memorial Sloan-Kettering Cancer Center, New York, New York, United States of America; 3 Expression Analysis Inc., Durham, North Carolina, United States of America; 4 Watson School of Biological Sciences, Cold Spring Harbor Laboratory, Cold Spring Harbor, New York, United States of America; 5 Howard Hughes Medical Institute, Chevy Chase, Maryland, United States of America; Roswell Park Cancer Institute, United States of America

## Abstract

There is significant need to identify novel prostate cancer drug targets because current hormone therapies eventually fail, leading to a drug-resistant and fatal disease termed castration-resistant prostate cancer. To functionally identify genes that, when silenced, decrease prostate cancer cell proliferation or induce cell death in combination with antiandrogens, we employed an RNA interference-based short hairpin RNA barcode screen in LNCaP human prostate cancer cells. We identified and validated four candidate genes (*AKT1*, *PSMC1*, *STRADA*, and *TTK*) that impaired growth when silenced in androgen receptor positive prostate cancer cells and enhanced the antiproliferative effects of antiandrogens. Inhibition of AKT with a pharmacologic inhibitor also induced apoptosis when combined with antiandrogens, consistent with recent evidence for PI3K and AR pathway crosstalk in prostate cancer cells. Recovery of hairpins targeting a known prostate cancer pathway validates the utility of shRNA library screening in prostate cancer as a broad strategy to identify new candidate drug targets.

## Introduction

Prostate cancer is the second leading cause of death from cancer in American men with an estimated 217,730 new cases and more than 32,000 deaths from the disease in 2010 [1]. While most early-stage localized disease can be successfully treated by radiation therapy and/or surgery, men who present with late-stage metastatic cancer have a median survival of 3–7 years [2]. Furthermore, as many as 50% of patients treated with localized disease will have local recurrence or distant metastases [2].

Current treatments for recurrent or metastatic disease include targeting androgen receptor (AR) signaling through the use of antiandrogens, such as bicalutamide, and drugs that prevent the production of androgens in the testicles and adrenal glands, such as gonadotropin-releasing hormone agonists and ketoconazole [3]. Although these current hormone therapies have initial effects in reducing tumor burden, many men become resistant to these therapies and develop castration-resistant prostate cancer (CRPC). Men with CPRC have a poor prognosis and account for the majority of deaths from the disease.

Men with CPRC often exhibit an increase in tumor androgen receptor (AR) levels [4]. AR is a 110 kDa nuclear hormone receptor that, in response to androgens, activates the transcription of target genes involved in cell proliferation, differentiation, and survival. In the prostatic luminal epithelium, AR regulates differentiation and proliferation, and AR in prostate cancer cells promotes cell cycle progression [5]. Previous work in our laboratory shows that this increased AR level is necessary and sufficient for the progression of prostate cancer to CRPC and its function is essential to sustain tumor growth [6]. In addition to CRPC, *AR* is expressed in nearly all prostate tumors and is required for tumor maintenance [4], [7], [8]. Taken together, these data suggest that AR signaling plays a critical role in hormone-sensitive and CRPC and remains an important target for prostate cancer therapeutics.

Identification of novel approaches to treat prostate cancer is an area of intense therapeutic development. Recently abiraterone acetate was approved based on a significant survival benefit in patients with CRPC, and the novel antiandrogens, MDV3100 and ARN-509, have been introduced with promising results; however most tumors acquired resistance to these therapeutics [9]–[13]. To date among chemotherapeutic agents only the taxanes docetaxel and cabazitaxel have been shown to improve overall survival in patients with CRPC [14]–[16]. As a result of the lack of agents that sustain prostate cancer regression, new prostate cancer therapeutic targets warrant further investigation.

To uncover potential prostate cancer therapeutic targets, we performed an unbiased multiplex shRNA screen that identified modulators of prostate cancer cell viability in the presence of bicalutamide. Four genes were validated to amplify the antiproliferative effects of anti-androgens in a prostate cancer cell line when silenced. These data provide a general strategy to identify prostate cancer drug targets.

## Results

### shRNA multiplex screen to identify modulators of bicalutamide sensitivity

**Figure 1:**
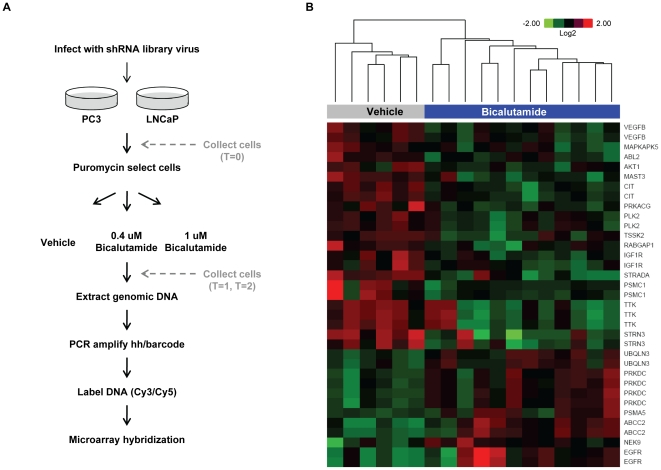
shRNA probes depleted or enriched in bicalutamide-treated LNCaP cells. (A) Schematic of shRNA screen. Details can be found in the Materials and Methods section. (B) A heatmap was generated by clustering based on probes that were depleted or enriched (log2 bicalutamide/vehicle ≤+/−0.58 and a p-value≤0.01) in the bicalutamide (0.4 uM and 1.0 uM)-treated LNCaP cells at T = 1 and T = 2 compared to the vehicle-treated cells at the same timepoints. The shRNA target gene associated with each probe is indicated to the right of the heatmap. Target genes that appear more than one time on the heatmap indicate that more than one probe scored for that gene.

In order to identify genes that, when silenced, reduce cell viability alone or in combination with the antiandrogen, bicalutamide, we utilized a multiplex RNA interference-based shRNA screen using a previously validated library (**Figure 1A**). This technology employs uniquely barcoded shRNAs, expressed from a retroviral vector, whose abundance after cell manipulation can be identified by microarray [17]. The library was comprised of ∼6,000 shRNAs targeting kinases, genes involved in cell cycle regulation, and other genes known to be involved in cancer [17]. Analysis of expression data from 147 prostate tumor samples [18] showed that 97% of the genes targeted by shRNAs in the library are detected in at least 50% of the tumors. We utilized the androgen receptor (AR)-positive LNCaP cell line for the screen because they undergo growth arrest when treated with the AR antagonist bicalutamide, grow relatively quickly, and are easily infected with retrovirus (**Figure S1**). AR-negative PC3 human prostate cancer cells served as a negative control of antiandrogen sensitivity (**Figure S1**). Correlation between biological replicate experiments in each cell line was high and did not change at later time points or with bicalutamide treatment (**Table S1**).

Microarray analyses revealed that 23 probes, associated with 15 genes, were uniquely depleted in bicalutamide-treated LNCaP cells, when compared to vehicle-treated cells (log2 bicalutamide/vehicle ≤−0.58, p≤0.01) (**Figure 1B** **,** **Table 1** **, and Figure S2**). No differences in depleted probes were observed across high and low bicalutamide doses or early and late timepoints (day 8 or day 21); therefore the data were combined for the analyses. Of the 15 genes identified, 11 were kinases (*TTK*, *MAST3*, *CIT*, *PRKACG*, *IGF1R*, *TSSK2*, *VEGFβ*, *MAPKAPK5*, *ABL2*, *PLK2*, and *AKT1*) known to have functions in cell cycle regulation or cell viability (**Table 1**). This high frequency of recovering kinase hits may be relevant to the biology of prostate cancer cells but also likely reflects a bias in the selection of genes for the shRNA library. The remaining 4 gene hits have disparate functions: calmodulin binding (*STRN3*), GTPase activation (*RABGAP1*), kinase adaptors (*STRADA*), or involved with the proteasome (*PSMC1*). A parallel screen was also performed in the androgen-independent cell line, PC3, and, notably, none of the probes depleted in LNCaP cells were depleted in PC3 cells in the presence of bicalutamide using the same cut-off, providing evidence for specificity in antiandrogen sensitive cells (**Table S2**).

**Table 1:** Probes depleted in bicalutamide-treated LNCaP cells.

shRNA target gene	Description	Probes scored*	log2 (Bic/Veh)**	p value
*STRN3*	Striatin, calmodulin binding protein 3, cell cycle protein	2	−1.527 −1.008	0.0037 0.0073
*STRADA*	STE20-related kinase adaptor alpha	1	−1.197	0.0003
*TTK*	Ser/Thr/Tyr kinase, cell cycle regulated	3	−1.014 −1.018 −0.991	0.0039 0.0040 0.0073
*MAST3*	Microtubule associated Ser/Thr kinase 3	1	−0.901	0.0009
*PSMC1*	Proteasome 26S subunit ATPase 1	2	−0.900 −0.880	0.0048 0.0060
*CIT*	Citron, Rho-interacting, Ser/Thr kinase 21	2	−0.802 −0.815	9.12E-05 0.0001
*RABGAP1*	GTPase-activating protein of RAB6A, microtubule/centrosome function	1	−0.791	0.0035
*PRKACG*	Gamma form of Protein Kinase A catalytic subunit	1	−0.758	0.0043
*IGF1R*	Insulin-like growth factor 1 receptor	2	−0.716 −0.681	0.0047 0.0091
*TSSK2*	Testes-specific serine kinase 2	1	−0.666	0.0045
*VEGFβ*	Vascular endothelial growth factor beta	2	−0.660 −0.594	0.0015 0.0026
*MAPKAPK5*	Mitogen-activated protein kinase-activated protein kinase 5	1	−0.645	0.0095
*ABL2*	V-abl Abelson murine leukemia viral oncogene homolog 2, tyrosine kinase	1	−0.621	0.0047
*PLK2*	Polo-like kinase 2, Ser/Thr kinase	2	−0.598 −0.584	0.0045 0.0085
*AKT1*	Ser/Thr kinase	1	−0.594	0.0041

* Number of probes that had a log2 value ≤−0.58.

** Bic, bicalutamide; Veh, vehicle.

### Validation of candidate genes

**Figure 2:**
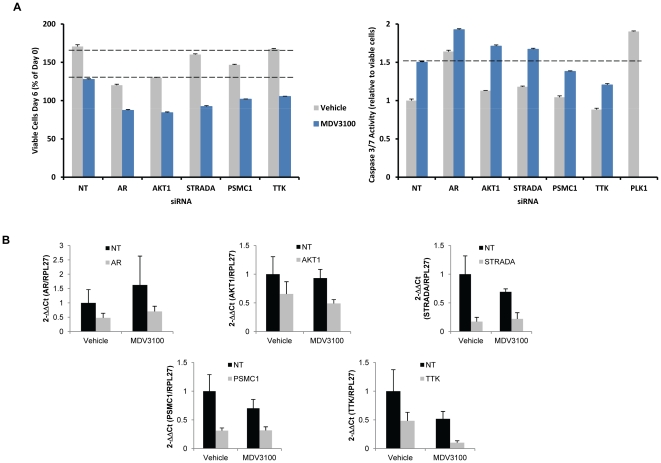
Silencing of a subset of genes inhibited VCaP proliferation and induced apoptosis. Candidate target genes from LNCaP screen probes that were depleted in the presence of bicalutamide were selectively targeted using siRNAs. (A, left panel), VCaP cells were transfected with siRNAs that scored in the LNCaP drug screen and were incubated with 1 uM MDV3100 or vehicle, and the number of viable cells was measured 6 days post-treatment. (A, right panel), VCaP cells were transfected and treated as in (A, left panel) except Caspase 3/7 activity was measured after 3 days of treatment. *PLK1* siRNA transfection was used as a positive control. Dashed lines indicate the level of growth inhibition or apoptosis induced by MDV3100, for comparison. NT, non-targeting siRNA. (B) Gene silencing (10–50%) was confirmed by RT-qPCR 6 days post-transfection of VCaP cells with the siRNA SMARTpools. Reactions were done in triplicate and normalized to RPL27 for each cDNA and then normalized to vehicle-treated NT. Standard error of the mean was calculated. Bic, bicalutamide.

To further examine these 15 candidate genes, we employed independent knockdown technology (siRNA transfection using siRNAs targeting sequences distinct from those used in the shRNA screen) in a second AR dependent cell line (VCaP), which has been shown to be resistant to bicalutamide but sensitive to the second generation antiandrogen MDV3100 [19]. Silencing of the genes was confirmed by qRT-PCR (**Figure 2B**). As a positive control, VCaP cells were transfected with AR siRNAs and, as expected, silencing of AR reduced cell viability (**Figure 2A**). Silencing of *AKT1*, *STRADA*, *PSMC1*, and *TTK* enhanced the growth inhibitory effect of MDV3100 in VCaP cells (**Figure 2A**, **left panel**), consistent with the effects observed with bicalutamide in the original screen in LNCaP cells. Interestingly, silencing *AKT1* and *PSMC1* in VCaP cells also decreased cell viability in the absence of antiandrogen (**Figure 2A** **, left panel**).

We then examined the effect of silencing *AKT1*, *STRADA*, *PSMC1*, and *TTK* on apoptosis, using *PLK1* siRNAs as a positive control. Silencing of *AR*, *AKT*, and *STRADA* in combination with MDV3100 treatment induced VCaP cell apoptosis over control siRNAs (NT) treated with MDV3100 (**Figure 2A** **, right panel**). With the exception of AR, none of the siRNAs tested induced apoptosis in the absence of MDV3100 (**Figure 2A** **, right panel**). Although silencing of *TTK* in combination with MDV3100 did not induce apoptosis over the NT cells with MDV3100, the combination did reduce the number of viable cells more than MDV3100 alone in the NT cells (**Figure 2A** **, left panel**). Taken together, *AKT*, *STRADA*, and *TTK* siRNAs synergize with MDV3100 to reduce VCaP cell viability. Whereas *AKT1* and *STRADA* silencing reduces cell viability, at least partially, due to increased apoptosis when combined with MDV3100, *TTK* seems to work through an alternative growth inhibitory mechanism. Although *PSMC1* did not score in the PC3 cells in the initial shRNA library screen, siRNA knockdown of *PSMC1* impaired viability of PC3 cells, raising the possibility that these antiproliferative effects may not be specific to AR-positive prostate cancer cells (**Figure S3**). For this reason, we did not pursue further characterization of *PSMC1*.

### Overexpression of *TTK* is associated with increased probability of biochemical recurrence

**Figure 3:**
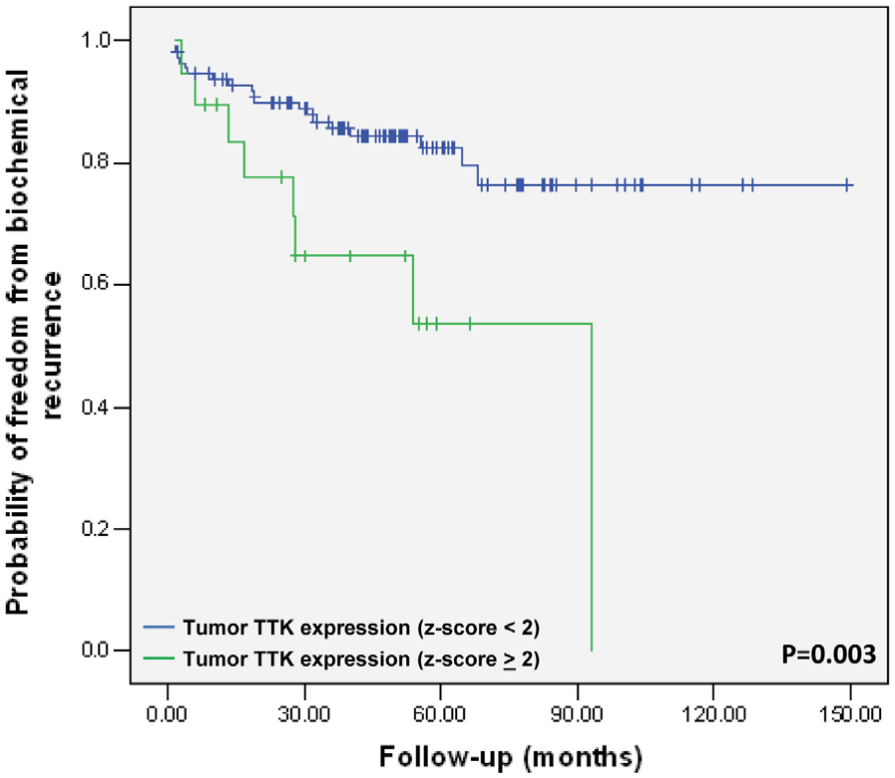
Higher *TTK* expression is associated with biochemical recurrence. Kaplan Meier plot of the risk of biochemical recurrence in patients (n = 131) with TTK overexpressing prostate tumors (n = 20; green line) versus those without TTK overexpressing (n = 111; blue line) tumors (p = 0.003, log-rank test).

To explore whether *AKT1*, *STRADA*, and *TTK* are altered in human prostate cancer, we assessed the copy number and expression status of these genes in a previously-reported human prostate cancer dataset [18]. Of the 218 prostate tumors, 34.4% had reduced expression of *STRADA*, 18.3% exhibited overexpression of *TTK*, 11.7% either overexpressed or had reduced expression of *AKT1*, and 15.6% of tumors overexpressed or had amplified *AR* (**Table 2**). Overexpression was defined as z-score≥2 and reduced expression was defined as z-score<2, compared to expression in normal prostate samples. Unlike *AR*, *STRADA*, *TTK*, and *AKT1* showed little evidence of gene amplification or loss in human prostate tumors. The large number of prostate tumors with alterations in *STRADA*, *TTK*, and *AKT1* expression led us to look at their correlation with biochemical recurrence. Interestingly, overexpression of *TTK* exhibited a significant association with biochemical recurrence (p = 0.003) (**Figure 3**). TTK expression did not significantly correlate with the following clinical characteristics: surgical margin, lymph node status, seminal vesicle, Gleason score, treatment prostate specific antigen (PSA), mean PSA, or extracapsular extension (**Table S3**).

**Table 2:** Candidate gene expression and alterations in human prostate tumors.

Gene	% of prostate tumors with alteration (n = 218)	mRNA Upregulation	mRNA Downregulation	Amplification	Deletion
*AR*	15.1%	18*	4	15	0
*AKT1*	11.9%	12	14	0	0
*STRADA*	36.2%	4	74	1	0
*TTK*	18.4%	33	4	0	3

* 4 of the samples with upregulated AR mRNA also had *AR* amplification.

### Blocking AKT cooperates with bicalutamide to induce LNCaP cell apoptosis

**Figure 4:**
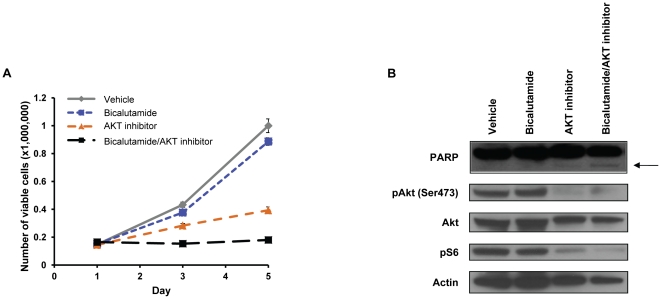
Akt1/2 inhibitor cooperated with bicalutamide to induce LNCaP cell apoptosis. (A) Number of viable LNCaP cells treated over 5 days with vehicle, bicalutamide (1 uM), Akt1/2 inhibitor (1 uM), or a combination of bicalutamide and AKT inhibitor. (B) Apoptosis was measured in LNCaP cells by immunoblotting using a PARP antibody in LNCaP cells treated as in (A). Inhibition of phosphorylated Akt (pAkt (Ser473)) and phosphorylated S6 (pS6) was also measured. Actin was used as a loading control.

To further investigate the potential role of these genes as therapeutic targets in prostate cancer, we turned to pharmacological inhibitors. We assessed the number of viable LNCaP cells using an AKT1/2 inhibitor in the presence and absence of bicalutamide. Treatment with bicalutamide or the AKT inhibitor reduced the number of viable cells by 10% and 45%, respectively (**Figure 4A**). In contrast, combining the compounds reduced cell proliferation by 100% and induced apoptosis as shown by an increase in PARP cleavage (**Figure 4B**). These data suggest that AKT may be a therapeutic target for prostate cancer in combination with antiandrogen therapy, consistent with recent evidence using PI3K inhibitors [20].

## Discussion

Current therapies for prostate cancer include antiandrogens such as bicalutamide and MDV3100. Although nearly all patients have initial responses to these drugs, many tumors become resistant to these therapies, resulting in CRPC. Due to the important role that AR plays in primary prostate cancer and CRPC, AR remains a relevant therapeutic target. Identification of novel approaches to treat prostate cancer, including the development of combination therapies with antiandrogens, is an area of therapeutic development. Therefore, we executed an unbiased multiplex shRNA screen to identify modulators of prostate cancer cell viability. Our goal was to identify genes that might be exploited for novel targeted therapy.

From the shRNA screen and *in vitro* siRNA validation experiments in an independent cell line, we identified and validated *AKT1*, *STRADA*, and *TTK* as candidate genes that synergize with antiandrogens (**Figure 1** **and** **Figure 2**). Although *AKT1*, *STRADA*, and *TTK* scored as inhibitors of LNCaP cell proliferation in the context of bicalutamide, validation with siRNAs in the same cell line revealed that they can decrease cell viability in the absence of bicalutamide (**Figure S4A**). This effect was specific to LNCaP and was not observed in AR-negative PC3 cells. One explanation for this difference in phenotype is the strength of shRNA knockdown in the screen versus the siRNA in the validation experiments, since siRNAs generally achieve greater knockdown than the shRNAs at an MOI≤0.5. Indeed, the siRNAs did exhibit a high level of gene silencing in the validation experiments (**Figure S4B**).

It will be of interest to understand the mechanism by which inhibition of *AKT1*, *STRADA* or *TTK* enhances antiandrogen activity. One possibility is through regulating AR-dependent transcription but we failed to observe a decrease in mRNA levels of *AR* or AR-target genes (*TMPRSS2*, *FKBP5*, *NKX3.1*, or *KLK3*) when these genes were silenced in LNCaP cells (data not shown). Another possibility is disruption of signaling pathway crosstalk, as illustrated by recent evidence for reciprocal negative feedback as an explanation for the synergy observed with combined PI3K/AR pathway inhibition [20].

TTK, also known as monopolar spindle 1 (Mps1), is a serine/threonine and tyrosine kinase that has been implicated in the maintenance of the spindle assembly checkpoint and is required for proper chromosome segregation during mitosis [21], [22]. TTK is of particular interest since others have reported that reducing levels of *TTK* sensitized human tumor cells to sub-lethal doses of taxol, whereas nontumorigenic cells could not be sensitized to taxol [23]. Here we find that that silencing *TTK* resulted in sensitization of VCaP cells to a potent antiandrogen. The fact that 18% of human prostate tumors overexpress TTK and that these tumors have a different clinical outcome suggests that TTK inhibitors in combination with antiandrogens may be of interest.

STE20-related kinase adaptor alpha, or STRADA, is a pseudokinase that is reported to be required for proper activity of the tumor suppressor, liver kinase B1 (LKB1) [24]. *STRADA* is required for function of the LKB1 tumor suppressor; therefore, silencing of *STRADA* might be expected to promote tumorigenesis. Our finding that *STRADA* exhibited reduced expression in a large percent of human prostate cancers is consistent with a tumor suppressor role (**Table 2**). However, it is possible that STRADA has functions independent of LKB1/AMPK signaling that promote growth inhibition in certain cellular contexts. The potential role of STRADA in prostate cancer progression and the possibility of LKB1/AMPK-independent functions warrant additional attention.

Akt1 is a serine-threonine kinase that relays signals from phosphatidylinositol 3′ kinase (PI3K) by its phosphorylation of downstream targets and deregulation of this pathway is known to contribute to prostate cancer progression [25], [26]. It is well known that the PI3K/Akt pathway plays an important role in prostate cancer cell viability and tumorigenesis and it is being currently being investigated as a therapeutic target [27], [28]. Previous reports have shown that the AKT/PI3K pathway regulates LNCaP cell growth and that expression of constitutively active AKT can block bicalutamide-induced growth inhibition [27], [29], [30]. Furthermore, Akt1 has been demonstrated to interact with AR [31]. Loss of function of the phosphatase and tensin homolog (PTEN), a negative regulator of the PI3K/AKT pathway, occurs in at least 50% of advanced human prostate cancer [32]–[34]. This loss of Pten function results in increased expression of phosphorylated Akt and subsequent activation of downstream targets involved in cell survival and proliferation [35], [36]. Inhibition of AKT using siRNAs or a pharmacological inhibitor induced apoptosis in combination with bicalutamide or MDV3100 in AR-dependent prostate cancer cells (**Figure 2A** **, right panel and** **Figure 4**). As a result of the reported roles of Akt1 in prostate cancer tumorigenesis, the PI3K/AKT pathway is being closely examined as a therapeutic target [25]. Our data support the use of Akt inhibitors in combination with antiandrogens to treat prostate cancer.

## Materials and Methods

### Cell culture and antiandrogens

Human prostate carcinoma cell lines (LNCaP, PC3, and VCaP) and human embryonic kidney cells (293T) were purchased from American Type Culture Collection (Manassas, VA) and were maintained below 20 passages in the laboratory. Cells were cultured according to ATCC guidelines. No abnormalities were observed in growth or morphology for any cell line under their respective growth conditions. Bicalutamide was purchased from AstraZeneca Pharmaceuticals LP (Wilmington, DE) and MDV3100 was synthesized at Memorial Sloan-Kettering Cancer Center by the Organic Synthesis Core Facility [19] .

### Preparation of short-hairpin RNA library DNA and virus

A plasmid library (pLMP) expressing ∼6,000 short-hairpin RNAs (shRNAs) targeting kinases, genes involved in cell cycle regulation, and other genes known to be involved in cancer was used in the current screen [17]. The pooled plasmid library was amplified in TOP10 electrocompetent cells (Invitrogen, Carlsbad, CA) and plasmid DNA was isolated from at least 1,000 transformants per hairpin. Virus was produced in 293FT cells by co-transfecting the pUMVC3-Gag-Pol expression vector (Addgene, Cambridge, MA), VSVG pantropic envelope (Addgene), and the shRNA library DNA isolated above.

### shRNA interference screen

The shRNA interference screen is depicted in **Figure 1A**. LNCaP and PC3 cells were infected with the 6K shRNA pooled library virus (MOI≤0.5) in triplicate to generate a total of 6 million infected cells per replicate, per cell line. Infection of target cells at MOI≤0.5 was confirmed by fluorescence microscopy and fluorescence activated cell sorting (FACS) 48 h after infection. LNCaP and PC3 cells were selected with 3 ug/ml puromycin and the cells were counted and plated in growth media containing 0.4 uM bicalutamide, 1.0 uM bicalutamide, or vehicle (DMSO). Cells were passaged, and bicalutamide and vehicle replenished, every 4 days without allowing confluence. LNCaP and PC3 cells from each replicate were harvested and cell pellets were flash frozen and stored at −80°C at multiple time-points during the screen. Cells were harvested 48 h after infection (T = 0), and after 8 days (T = 1) and 21 days (T = 2) of exposure to vehicle or bicalutamide.

### Microarray

Genomic DNA was extracted from cells using standard conditions. Barcodes and half-hairpins from the library plasmid DNA (originally used to make the virus) and LNCaP and PC3 cell genomic DNA isolated above were PCR amplified using the following primers: 5′-TAGTGAAGCCACAGATGTA-3′ and 5′-AAAGCGCATGCTCCAGACTGCC-3′. The PCR products were subject to gel electrophoresis and the resulting 350 bp bands were gel purified using the QIAEX II gel extraction kit (QIAGEN, Valencia, CA).Purified DNA fragments (1.5 ug) from LNCaP or PC3 cells were labeled and microarray hybridization was carried out using custom Agilent chips as previously described [17].

### Microarray data analysis

Affymetrix exon array data from 147 tumor samples [18] were used to determine expression levels of shRNA target genes represented in the library screen. Probes with background adjusted normalized intensity greater than 50 were considered as detected.

Agilent Feature Extractor software was used to scan microarray images. Feature Extractor normalized Cy3 intensity for each array was compiled and all further processing was performed using the statistical software package R 2.8.1. Quality was assessed throughout using principal component analysis and more formally by comparing the interquartile range across arrays. Samples with a log2 transformed interquartile range less than 6 were considered outliers and not considered in further analysis. When duplicate samples (technical replicates) remain, the most recent replicate of each was used. The remaining samples were subject to quantile normalization. Probes corresponding to hairpins not present in the library were used as negative controls. The 95th percentile signal intensity of negative controls was used as the background measure and was estimated from pre-selection (T = 0) samples. Probes were removed from further analysis when they did not exceed the sample specific background estimate on a majority of pre-selection samples. Statistical testing was performed to identify hairpins whose abundance varied over time in untreated samples. The time course analysis implemented in the extraction of differential gene expression (EDGE) software was used for this analysis [37]. To identify hairpins where abundance was associated with the presence of bicalutamide the linear models for microarray library in R 2.8.1 was used [38]. Specifically, a linear model was constructed with terms for treatment, time point, and replicate. This model was fit to each probe, and fold changes and p-values corresponding to the treatment effect were used to identify probes of interest. The two bicalutamide doses (0.4 uM and 1.0 uM) and the two timepoints collected post-selection (T = 1 and T = 2) were combined for the analysis to increase statistical power. Significant differences between bicalutamide doses and timepoints were not observed. Candidates that had an abundance change that was associated with the presence of bicalutamide were selected that resulted in a log2 bicalutamide/vehicle ≤−0.58 and a p-value≤0.01. Visualization of candidates was performed with hierarchical clustering and heatmaps. All microarray data is MIAME compliant. All raw data has been deposited in GEO (series ID GSE32261).

### Cell viability and apoptosis assays

ON-TARGETplus non-targeting (NT) siRNA and siRNA SMARTpools targeting *ABL2*, *AKT1*, *AR*, *CIT*, *IGF1R*, *MAPKAPK5*, *MAST3*, *PLK2*, *PRKACG*, *PSMC1*, *RABGAP1*, *STRADα (STRADA)*, *STRN3*, *TSSK2*, *TTK*, and *VEGFβ* were purchased from Thermo Fisher Scientific (Lafayette, CO). siRNAs targeting *PLK1* were used as a positive control for apoptosis and were obtained from the MSKCC High-Throughput Drug Screening Facility. Log phase LNCaP, PC3, and VCaP cells were transfected with 100 nM siRNA using DharmaFECT (Thermo Fisher Scientific). Cell viability was determined 3 and 6 days after incubation with bicalutamide (1 uM), MDV3100 (1 uM), or vehicle using the Cell Titer-Glo Luminescent cell viability assay (Promega). Apoptosis was assayed using the Caspase-Glo 3/7 assay (Promega) and values were normalized to the Day 3 Cell Titer-Glo assay. Assays were performed in triplicate and standard errors of the mean are reported. Cell proliferation assays were also performed by incubating cells with vehicle (DMSO), bicalutamide (1 uM), AKT inhibitor (1 uM) (MERCK, Whitehouse Station, NJ), or a combination of bicalutamide and AKT inhibitor and counting the cells. Experiments were conducted in triplicate and the standard deviations are reported.

### Immunoblotting

Cell lysates for Western blot analyses were prepared using standard RIPA buffer. The antibodies used for western blot analysis and immunohistochemistry were pAkt Ser473 (Cell Signaling Technology, 1∶1000 dilution), Akt (Cell Signaling Technology, Danvers, MA, 1∶1000 dilution), pS6 Ser235/236 (Cell Signaling Technology, 1∶1000 dilution), PARP (Cell Signaling Technology, 1∶1000 dilution), and Actin (Cell Signaling Technology, 1∶1000 dilution).

### Quantitative reverse transcription-PCR

LNCaP, PC3, and VCaP cells subject to siRNA transfection were harvested 4 days or 7 days post-transfection to monitor knockdown of the target gene(s) by quantitative reverse transcription-PCR (qRT-PCR) (see **Table S4**). Reactions were done in triplicate and normalized to RPL27 for each cDNA and then normalized to vehicle-treated NT. Standard error of the mean was calculated.

### Biochemical recurrence

The gene expression and copy number status of *STRADA*, *TTK*, *AR*, *AKT1*, *SKT22B*, *PSMC1*, and *PRKACG* in primary and metastatic human prostate cancer (n = 218) was determined by using the MSKCC Cancer Genomics Pathway Portal [18]. Twenty of the 131 tumors had *TTK* over-expression defined as z-score≥2. Cases were classified as *TTK* high and *TTK* normal/low. The probability of freedom from biochemical recurrence following radical prostatectomy (BCR defined as >0.2 ng/ml and rising prostate specific antigen) was reported using Kaplan-Meier analysis. P-value was calculated using the log-rank test.

## Supporting Information

Figure S1 **Bicalutamide inhibited LNCaP cell proliferation.** After infection with the shRNA library and puromycin selection (A) LNCaP and (B) PC3 cells were counted and plated in growth media containing 0.4 uM bicalutamide, 1.0 uM bicalutamide, or vehicle (0 uM). Cells were counted and passaged every 4 days to monitor growth in response to vehicle and bicalutamide. Results are presented as the average number of viable cells (top panels) or as the percent of viable bicalutamide-treated cells compared to vehicle-treated cells (bottom panels) at each time point over the 21 day time course for each drug treatment ± standard error of 3 replicate experiments.
(TIF)

Figure S2 **shRNA probes depleted in LNCaP cells.** Boxplots of the relative abundance of probes in the vehicle and bicalutamide-(drug) treated LNCaP cells. Data from T = 2 and T = 3 were combined for the vehicle or bicalutamide-treated boxplots. Both bicalutamide doses (0.4 uM and 1.0 uM) were also combined for the drug boxplots. The name of the shRNA probes appear in parentheses next to the target gene name above each boxplot.
(TIF)

Figure S3 **Silencing of PSMC1 inhibited PC3 cell proliferation.** (A) PC3 cells were transfected with siRNAs, treated with 1 uM bicalutamide or vehicle, and the number of viable cells was measured 6 days post-treatment. Only siRNAs that inhibited VCaP cell proliferation from Figure 2 are shown. The dashed line indicates the level of growth inhibition induced by bicalutamide, for comparison. (B) Gene silencing was confirmed by RT-qPCR 6 days post-transfection of VCaP cells with the siRNA SMARTpools. Reactions were done in triplicate and normalized to RPL27 for each cDNA and then normalized to vehicle-treated NT. Standard error of the mean was calculated. Bic, bicalutamide.
(TIF)

Figure S4 **Silencing of a subset of genes inhibited LNCaP proliferation.** Candidate target genes from LNCaP screen probes that were depleted in the presence of bicalutamide were selectively targeted using siRNAs. (A) LNCaP cells were transfected with siRNAs, treated with 1 uM bicalutamide or vehicle, and the number of viable cells was measured 6 days post-treatment. Only siRNAs that inhibited LNCaP cell proliferation are shown. The dashed line indicates the level of growth inhibition induced by bicalutamide, for comparison. (B) Gene silencing was confirmed by RT-qPCR 6 days post-transfection of LNCaP cells with the siRNA SMARTpools. Reactions were done in triplicate and normalized to RPL27 for each cDNA and then normalized to vehicle-treated NT. Standard error of the mean was calculated. Bic, bicalutamide. NT, non-targeting siRNA.
(TIF)

Table S1 **Correlation among replicate experiments.** Correlation between biological replicate microarray experiments in the LNCaP and PC3 cells 48 h after infection or after culture for 8 or 21 days in vehicle or bicalutamide. For the correlation analysis 0.4 uM and 1.0 uM bicalutamide doses were combined.
(DOCX)

Table S2 **Probes depleted in bicalutamide-treated PC3 cells.** The number of microarray probes that had a log2 value ≤−0.58 (probes scored) in the PC3 cells. Bic, bicalutamide; Veh, vehicle
(DOCX)

Table S3 **Tumor** ***TTK*** **expression correlation with clinical characteristics.** *TTK* expression in 131 prostate tumors and its correlation with surgical margin, lymph node status, seminal vesicle, Gleason score, treatment prostate specific antigen (PSA), mean PSA, or extracapsular extension. TTK overexpressed samples were defined as z-score≥2 and TTK not overexpressed samples were defined as z-score<2.
(DOCX)

Table S4 **Primers used for quantitative reverse transcription-PCR.** Primers were used for validation of the mRNA expression levels of the genes defined in Table 1.
(DOCX)
