# Supplementary figures and images for: Modulators of Prostate Cancer Cell Proliferation and Viability Identified by Short-Hairpin RNA Library Screening
Source: PLoS One. 2012 Apr 11;7(4):e34414. doi: 10.1371/journal.pone.0034414 (PMC3324507; doi:10.1371/journal.pone.0034414)

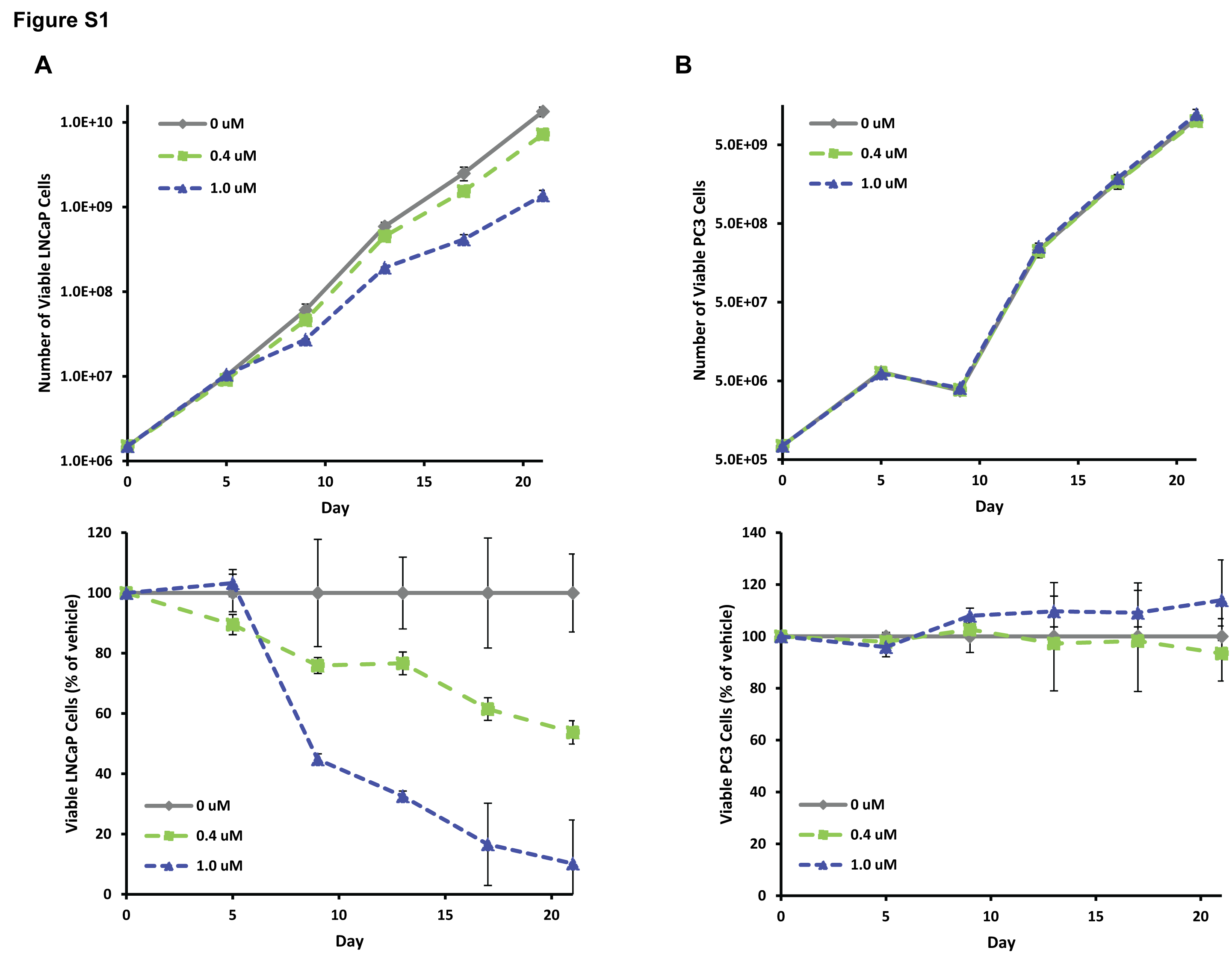

Supplement: Figure S1 — Bicalutamide inhibited LNCaP cell proliferation. After infection with the shRNA library and puromycin selection (A) LNCaP and (B) PC3 cells were counted and plated in growth media containing 0.4 uM bicalutamide, 1.0 uM bicalutamide, or vehicle (0 uM). Cells were counted and passaged every 4 days to monitor growth in response to vehicle and bicalutamide. Results are presented as the average number of viable cells (top panels) or as the percent of viable bicalutamide-treated cells compared to vehicle-treated cells (bottom panels) at each time point over the 21 day time course for each drug treatment ± standard error of 3 replicate experiments. (TIF) [file pone.0034414.s001.tif]

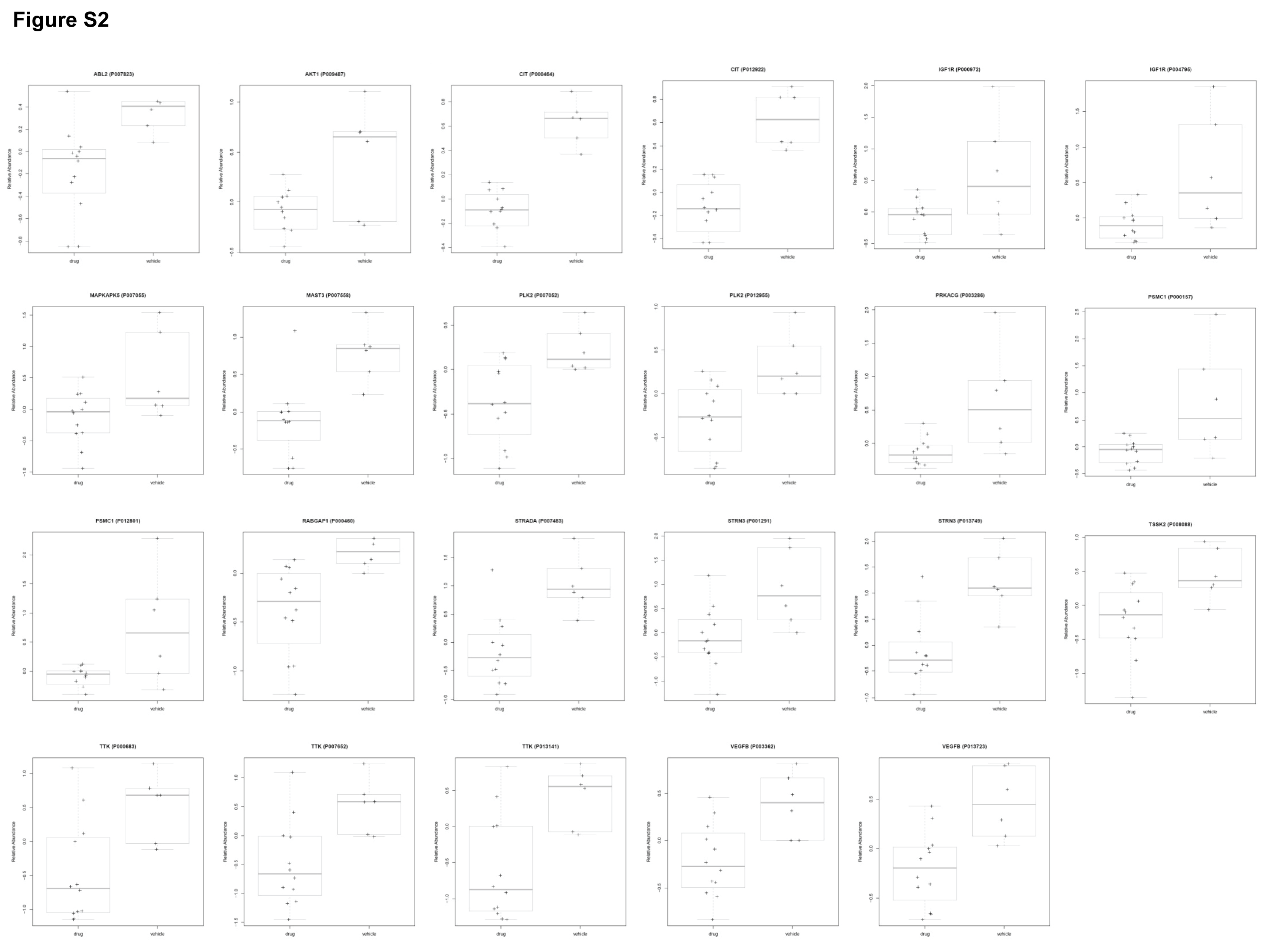

Supplement: Figure S2 — shRNA probes depleted in LNCaP cells. Boxplots of the relative abundance of probes in the vehicle and bicalutamide-(drug) treated LNCaP cells. Data from T = 2 and T = 3 were combined for the vehicle or bicalutamide-treated boxplots. Both bicalutamide doses (0.4 uM and 1.0 uM) were also combined for the drug boxplots. The name of the shRNA probes appear in parentheses next to the target gene name above each boxplot. (TIF) [file pone.0034414.s002.tif]

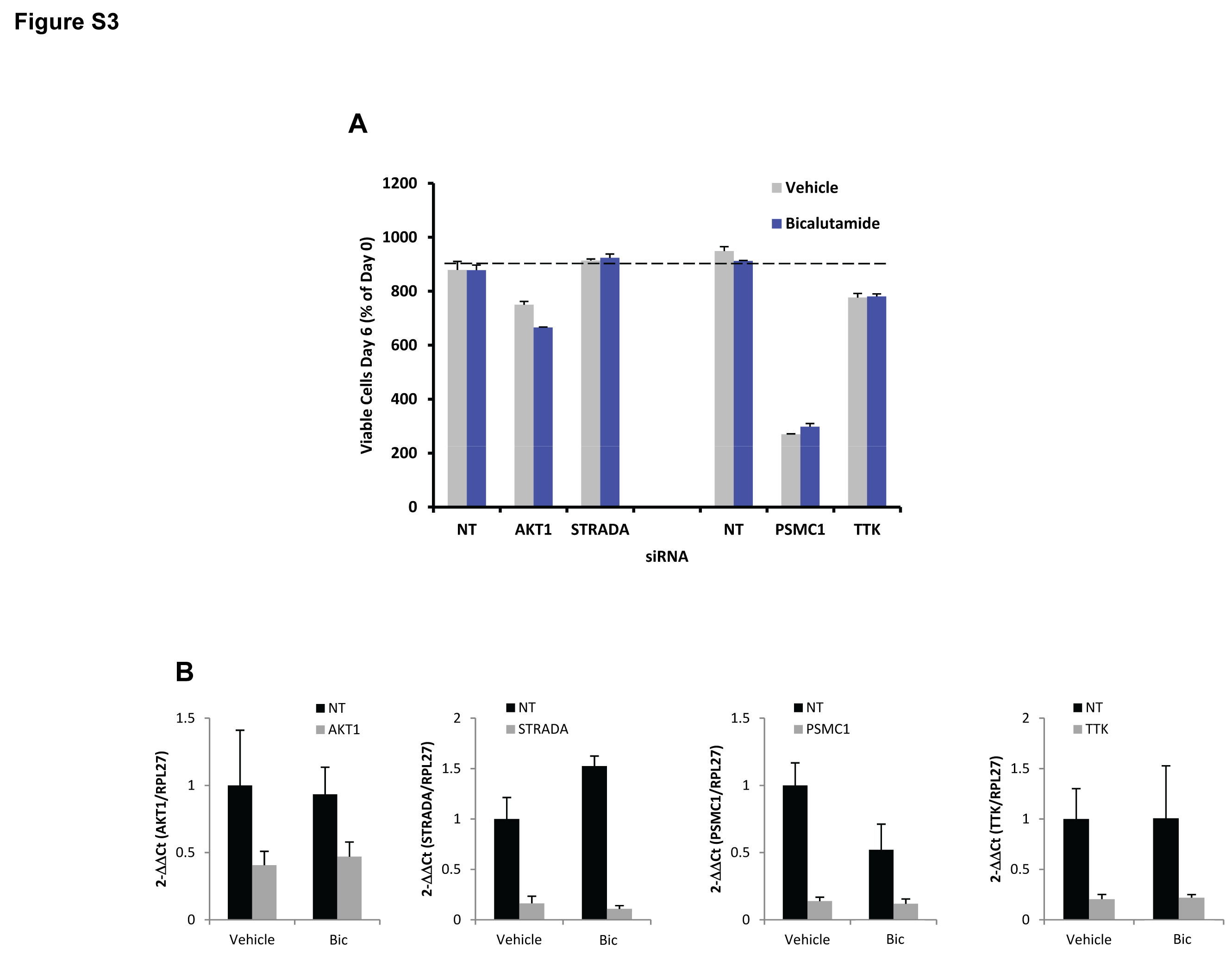

Supplement: Figure S3 — Silencing of PSMC1 inhibited PC3 cell proliferation. (A) PC3 cells were transfected with siRNAs, treated with 1 uM bicalutamide or vehicle, and the number of viable cells was measured 6 days post-treatment. Only siRNAs that inhibited VCaP cell proliferation from Figure 2 are shown. The dashed line indicates the level of growth inhibition induced by bicalutamide, for comparison. (B) Gene silencing was confirmed by RT-qPCR 6 days post-transfection of VCaP cells with the siRNA SMARTpools. Reactions were done in triplicate and normalized to RPL27 for each cDNA and then normalized to vehicle-treated NT. Standard error of the mean was calculated. Bic, bicalutamide. (TIF) [file pone.0034414.s003.tif]

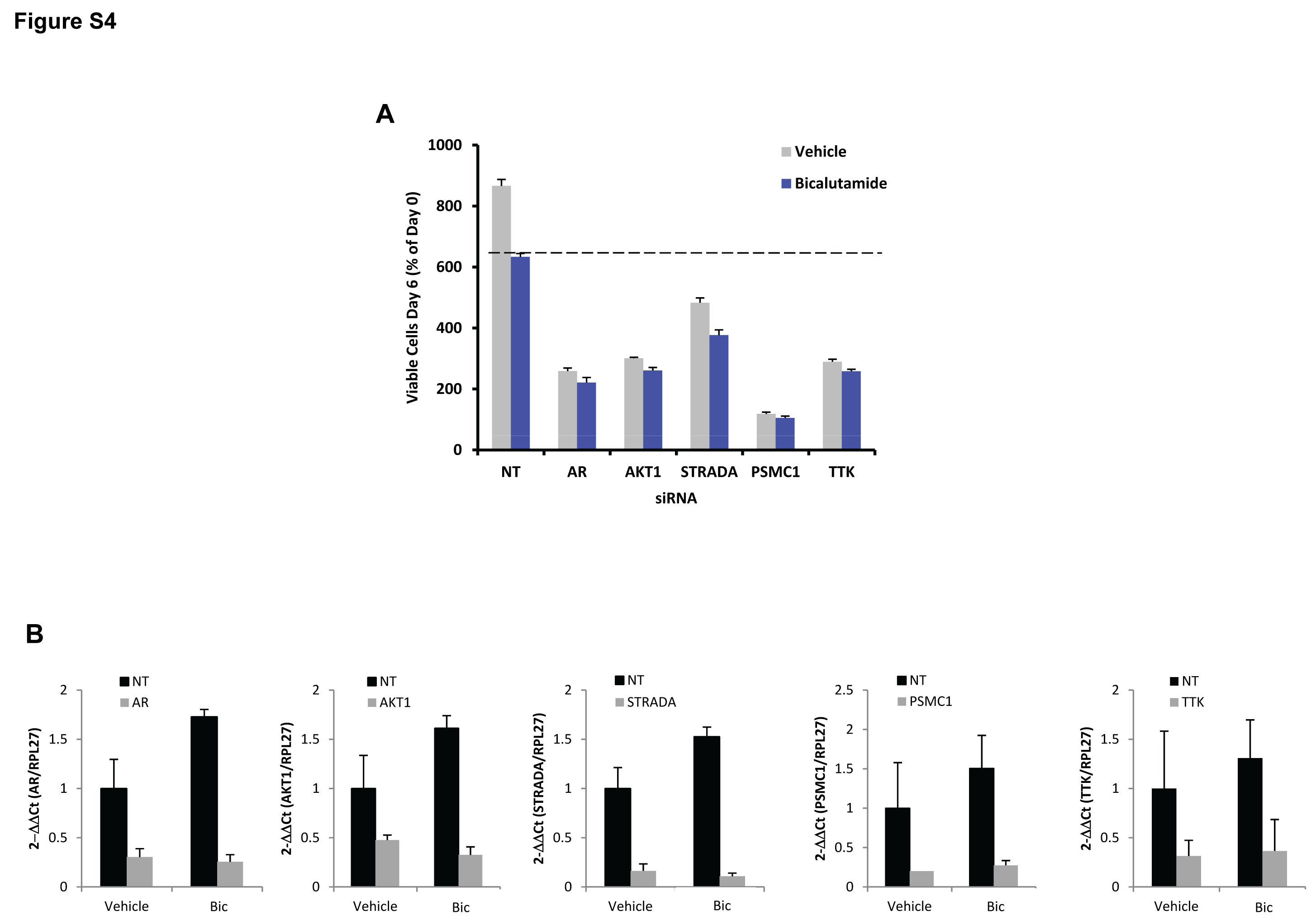

Supplement: Figure S4 — Silencing of a subset of genes inhibited LNCaP proliferation. Candidate target genes from LNCaP screen probes that were depleted in the presence of bicalutamide were selectively targeted using siRNAs. (A) LNCaP cells were transfected with siRNAs, treated with 1 uM bicalutamide or vehicle, and the number of viable cells was measured 6 days post-treatment. Only siRNAs that inhibited LNCaP cell proliferation are shown. The dashed line indicates the level of growth inhibition induced by bicalutamide, for comparison. (B) Gene silencing was confirmed by RT-qPCR 6 days post-transfection of LNCaP cells with the siRNA SMARTpools. Reactions were done in triplicate and normalized to RPL27 for each cDNA and then normalized to vehicle-treated NT. Standard error of the mean was calculated. Bic, bicalutamide. NT, non-targeting siRNA. (TIF) [file pone.0034414.s004.tif]
