# Supplementary material for: Modulators of Prostate Cancer Cell Proliferation and Viability Identified by Short-Hairpin RNA Library Screening
Source: PLoS One. 2012 Apr 11;7(4):e34414. doi: 10.1371/journal.pone.0034414 (PMC3324507; doi:10.1371/journal.pone.0034414)
Supplement: Table S1 — Correlation among replicate experiments. Correlation between biological replicate microarray experiments in the LNCaP and PC3 cells 48 h after infection or after culture for 8 or 21 days in vehicle or bicalutamide. For the correlation analysis 0.4 uM and 1.0 uM bicalutamide doses were combined. (DOCX) [file pone.0034414.s005.docx]

**Table S1. Correlation among replicate experiments.**

| **Group^*^** | **Correlation**^**^ | **Standard Error of the Mean** |
| --- | --- | --- |
|  |  |  |
| LNCaP T=1 (Day 8) vehicle | 0.983 | 0.0012 |
|  |  |  |
| LNCaP T=2 (Day 21) vehicle | 0.984 | 0.0018 |
|  |  |  |
| LNCaP T=1 (Day 8) bicalutamide | 0.985 | 0.0004 |
|  |  |  |
| LNCaP T=2 (Day 21) bicalutamide | 0.972 | 0.0028 |
|  |  |  |
| PC3 T=1 (Day 8) vehicle | 0.987 | 0.0010 |
|  |  |  |
| PC3 T=2 (Day 21) vehicle | 0.982 | 0.0030 |
|  |  |  |
| PC3 T=1 (Day 8) bicalutamide | 0.969 | 0.0032 |
|  |  |  |
| PC3 T=2 (Day 21) bicalutamide | 0.981 | 0.0008 |
|  |  |  |

^*^LNCaP or PC3 microarray data from cells 48 h after infection or after culture for 8 or 21 days in vehicle or bicalutamide. For the correlation analysis 0.4 uM and 1.0 uM bicalutamide doses were combined.

^**^Average of the correlation between the same probes of the three replicate experiments.
