# Supplementary material for: Modulators of Prostate Cancer Cell Proliferation and Viability Identified by Short-Hairpin RNA Library Screening
Source: PLoS One. 2012 Apr 11;7(4):e34414. doi: 10.1371/journal.pone.0034414 (PMC3324507; doi:10.1371/journal.pone.0034414)
Supplement: Table S2 — Probes depleted in bicalutamide-treated PC3 cells. The number of microarray probes that had a log2 value ≤−0.58 (probes scored) in the PC3 cells. Bic, bicalutamide; Veh, vehicle (DOCX) [file pone.0034414.s006.docx]

**Table S2. Probes depleted in bicalutamide-treated PC3 cells.**

| **shRNA target gene** | **Description** | **Probes scored^*^** | **log2 (Bic/Veh)^**^** | **p value** |
| --- | --- | --- | --- | --- |
| *MAP2K5* | Mitogen-activated protein kinase kinase 5 | 2 | -1.644  -1.651 | 0.0014  0.0018 |
| *CDK12* | Cyclin-dependent kinase 12 | 1 | -1.376 | 0.0022 |
| *CAMKK1* | Calcium/calmodulin-dependent protein kinase kinase 1, alpha | 1 | -1.347 | 0.0088 |
| *PTPRT* | Protein tyrosine phosphatase, receptor type, T | 1 | -1.138 | 0.0047 |
| *PSMA3* | Proteasome subunit, alpha type, 3 | 2 | -1.066 -1.017 | 0.0012 0.0020 |
| *PAK3* | p21 (CDKN1A)-activated kinase 3 | 1 | -1.027 | 0.0057 |
| *PTPRR* | Protein tyrosine phosphatase, receptor type, R | 3 | -0.851  -0.810  -0.698 | 0.0008  0.0011  0.0054 |
| *PSMD7* | Proteasome 26S subunit, non-ATPase, 7 | 1 | -0.847 | 0.0015 |
| *IL8* | Interleukin 8 | 2 | -0.791 -0.683 | 0.0045 0.0051 |
| *CSNK2A2* | Casein kinase 2, alpha prime polypeptide | 1 | -0.785 | 0.0052 |
| *YES1* | Yamaguchi sarcoma viral oncogene homolog 1 | 1 | -0.697 | 0.0068 |
| *PSMC2* | Proteasome 26S subunit, ATPase, 2 | 2 | -0.675  -0.623 | 0.0037  0.0074 |
| *PVRL1* | Poliovirus receptor-related 1 | 1 | -0.632 | 0.0016 |
| *SYK* | Spleen tyrosine kinase | 1 | -0.626 | 0.0017 |
| *ATR* | Ataxia telangiectasia and Rad3 related | 2 | -0.624 -0.596 | 0.0005 0.0007 |
| *HDAC4* | Histone deacetylase 4 | 1 | -0.612 | 0.0064 |
| *IL16* | Interleukin 16 | 1 | -0.595 | 0.0033 |
| *SLC8A1* | Solute carrier family 8, member 1, transcript variant A | 1 | -0.595 | 0.0049 |
| *PMS2* | Postmeiotic segregation increased 2 | 1 | -0.594 | 0.0008 |

^*^Number of probes that had a log2 value < - 0.58

^**^Bic, bicalutamide; Veh, vehicle
