# Supplementary material for: Modulators of Prostate Cancer Cell Proliferation and Viability Identified by Short-Hairpin RNA Library Screening
Source: PLoS One. 2012 Apr 11;7(4):e34414. doi: 10.1371/journal.pone.0034414 (PMC3324507; doi:10.1371/journal.pone.0034414)
Supplement: Table S3 — Tumor TTK expression correlation with clinical characteristics. TTK expression in 131 prostate tumors and its correlation with surgical margin, lymph node status, seminal vesicle, Gleason score, treatment prostate specific antigen (PSA), mean PSA, or extracapsular extension. TTK overexpressed samples were defined as z-score≥2 and TTK not overexpressed samples were defined as z-score<2. (DOCX) [file pone.0034414.s007.docx]

**Table S3. Tumor *TTK* expression correlation with clinical characteristics.**

| **Clinical Characteristic** | | **TTK Expression*** | | **p value** |
| --- | --- | --- | --- | --- |
|  |  | **Not overexpressed** | **Overexpressed** |  |
|  |  |  |  |  |
| Surgical Margin | Negative | 87 (78%) | 13 (65%) | 0.195 |
|  | Positive | 24 (22%) | 7 (35%) |  |
|  |  |  |  |  |
| Lymph Node | Negative | 85 (94%) | 17 (94%) | 1.000 |
|  | Positive | 5 (6%) | 1 (6%) |  |
|  |  |  |  |  |
| Seminal Vesicle | Negative | 99 (89%) | 18 (90%) | 0.914 |
|  | Positive | 12 (11%) | 2 (10%) |  |
|  |  |  |  |  |
| Gleason Score | 6 | 37 (33%) | 4 (20%) | 0.092 |
|  | 7 | 64 (58%) | 11 (55%) |  |
|  | >8 | 10 (9%) | 5 (25%) |  |
|  |  |  |  |  |
| Treatment PSA | <4 | 17 (15%) | 5 (26%) | 0.083 |
|  | 4-10 | 71 (64%) | 7 (37%) |  |
|  | >10 | 23 (21%) | 7 (37%) |  |
|  |  |  |  |  |
| Mean PSA |  | 7.5 | 11.3 | 0.047 |
|  |  |  |  |  |
| Extracapsular Extension | None | 37 (33%) | 5 (25%) | 0.688 |
|  | Invade | 44 (40%) | 8 (40%) |  |
|  | Established | 30 (27%) | 7 (35%) |  |
|  |  |  |  |  |

*TTK overexpressed samples were defined as z-score >2 and TTK not overexpressed samples were defined as z-score <2.
