# Supplementary material for: Modulators of Prostate Cancer Cell Proliferation and Viability Identified by Short-Hairpin RNA Library Screening
Source: PLoS One. 2012 Apr 11;7(4):e34414. doi: 10.1371/journal.pone.0034414 (PMC3324507; doi:10.1371/journal.pone.0034414)
Supplement: Table S4 — Primers used for quantitative reverse transcription-PCR. Primers were used for validation of the mRNA expression levels of the genes defined in Table 1. (DOCX) [file pone.0034414.s008.docx]

**Table S4. Primers used for quantitative reverse transcription-PCR.**

| **Target gene** | **Forward/Reverse^*^** | **Primer sequence (5’ -> 3’)** |
| --- | --- | --- |
|  |  |  |
| *ABL2* | Forward | CCGGCTTCAATATCTTCACC |
|  | Reverse | CTGCCTCCAGTCTTGTCTCC |
|  |  |  |
| *AKT1* | Forward | AACACCATGGACAGGGAGAG |
|  | Reverse | CAGCCCCTTTGACTTCTTTG |
|  |  |  |
| *AR* | Forward | GCAGGAAGCAGTATCCGAAG |
|  | Reverse | GACACCGACACTGCCTTACA |
|  |  |  |
| *CIT* | Forward | ACCACCTCCCACTCAGTGTC |
|  | Reverse | AAAGTCACAGTTTCGTGGGG |
|  |  |  |
| *IGF1R* | Forward | AACAAAGCTGGGATACGGTG |
|  | Reverse | CAAACTGCTGACCAGCAAAA |
|  |  |  |
| *MAPKAPK5* | Forward | GTACTGGAGGCGCAAAGAAG |
|  | Reverse | GATAGTCCGGCTGTGGTGTT |
|  |  |  |
| *MAST3* | Forward | GCTTTGCCTTGCAACTTTTC |
|  | Reverse | CCGTAAAGTCATGCCTGGAT |
|  |  |  |
| *PLK2* | Forward | GGGACTCTTGGCAGCTGTAG |
|  | Reverse | TTGGTGACCCACTGAAATGA |
|  |  |  |
| *PRKACG* | N/A** | N/A** |
|  |  |  |
| *PSMC1* | Forward | CTGTGGAGCTTCCTCTCACC |
|  | Reverse | GCCAGGTGGACCATAGAGAA |
|  |  |  |
| *RABGAP1* | Forward | CAGCAAGATTGCACTACGGA |
|  | Reverse | TCTGTTTGGTCATCAGCAGC |
|  |  |  |
| *STRADA* | Forward | 9GTGGGTCTCGGAAAAGTTCA |
|  | Reverse | GAGCTCGCATCATTGGTTTT |
|  |  |  |
| *STRN3* | Forward | AGCTGCTGACCTAACTGACGA |
|  | Reverse | AACGTTATTGGTTCAGCCCA |
|  |  |  |
| *TSSK2* | N/A** | N/A** |
|  |  |  |
| *TTK* | Forward | CAGCAGCAACAGCATCAAAT |
|  | Reverse | TGCTTGAACCTCCACTTCCT |
|  |  |  |
| *VEGFβ* | Forward | AGGCCATCATCAAACAGGAC |
|  | Reverse | TGCAGAGGTTTGGGTCTTCT |
|  |  |  |

^*^Direction of the primer

^**^Primers amplifying the target gene were not identified
